# Young Adult Cancer Survivorship: Recommendations for Patient Follow-up, Exercise Therapy, and Research

**DOI:** 10.1093/jncics/pkaa099

**Published:** 2020-10-28

**Authors:** Scott C Adams, Jennifer Herman, Iliana C Lega, Laura Mitchell, David Hodgson, Kim Edelstein, Lois B Travis, Catherine M Sabiston, Paaladinesh Thavendiranathan, Abha A Gupta

**Affiliations:** 1 Department of Cardiology, Toronto General Hospital Research Institute, Toronto, ON, Canada;; 2 Ted Rogers Cardiotoxicity Prevention Program, Peter Munk Cardiac Centre, Toronto General Hospital, Toronto, ON, Canada;; 3 Mental Health & Physical Activity Research Centre, Faculty of Kinesiology & Physical Education, University of Toronto, Toronto, ON, Canada;; 4 Adolescent and Young Adult Oncology Program, Department of Supportive Care, Princess Margaret Cancer Centre, Toronto, ON, Canada;; 5 Women’s College Hospital, University of Toronto, Toronto, ON, Canada;; 6 Division of Radiation Oncology, Princess Margaret Cancer Centre, Toronto, ON, Canada;; 7 Department of Radiation Oncology, University of Toronto, Toronto, ON, Canada;; 8 Department of Supportive Care, Princess Margaret Cancer Centre, Toronto, ON, Canada;; 9 Department of Psychiatry, University of Toronto, Toronto, ON, Canada;; 10 Division of Medical Oncology, Melvin and Bren Simon Cancer Centre, Indiana University, Indianapolis, IN, USA; 11 Department of Epidemiology, Fairbanks School of Public Health, Indiana University, Indianapolis, IN, USA;; 12 Department of Medicine, University of Toronto, Toronto, ON, Canada;; 13 Haematology/Oncology, The Hospital for Sick Children, Toronto, ON, Canada; 14 Department of Paediatrics, University of Toronto, Toronto, ON, Canada

## Abstract

Survivors of adolescent and young adult cancers (AYAs) often live 50 to 60 years beyond their diagnosis. This rapidly growing cohort is at increased risk for cancer- and treatment-related ‘late effects’ that persist for decades into survivorship. Recognition of similar issues in pediatric cancer survivors has prompted the development of evidence-based guidelines for late effects screening and care. However, corresponding evidence-based guidelines for AYAs have not been developed. We hosted an AYA survivorship symposium for a large group of multidisciplinary AYA stakeholders (approximately 200 were in attendance) at Princess Margaret Cancer Centre (Toronto, Ontario, Canada) to begin addressing this disparity. The following overview briefly summarizes and discusses the symposium’s stakeholder-identified high-priority targets for late effects screening and care and highlights knowledge gaps to direct future research in the field of AYA survivorship. This overview, although not exhaustive, is intended to stimulate clinicians to consider these high-priority screening and care targets when seeing survivors in clinical settings and, ultimately, to support the development of evidence-based late effects screening and care guidelines for AYAs.

Adolescent and young adult cancer survivors (AYAs) have unique support needs often overlooked by existing pediatric and adult oncology care models (1). AYAs are at increased risk of developing cancer- and treatment-related ‘late effects’ (2,3), including secondary cancers (4,5), cardiovascular disease (CVD) (6), impaired cardiorespiratory fitness (CRF; eg, VO_2peak_) (7), endocrine dysfunction (8), fatigue (9), cognitive impairments (10, 11), and psychological distress (12). Current limitations in preventing and treating these sequelae likely contribute to increased suffering and disability (3), health care utilization and cost (13), and mortality risk (6) in AYAs. Recognition of similar issues in pediatric cancer survivors led to the establishment of evidence-based care guidelines. Health-care practitioners caring for AYAs have traditionally relied on guidelines developed for late effect screening and care in pediatric and older adult cancer survivors (8,14-17), until the recent publication of preliminary AYA oncology-focused survivorship guidelines (18). Although commendable, the authors of these guidelines were similarly forced to rely on indirect evidence from younger and older cancer survivors or expert opinion because of the scarcity of AYA-specific evidence. To help bring attention to and discuss this unresolved disparity, the AYA Program at the Princess Margaret Cancer Centre (Toronto, Ontario, Canada) hosted a 2-day meeting titled The AYA Survivorship Symposium: A New Vision (March 2019). This symposium brought together a multidisciplinary group of AYA cancer stakeholders (approximately 200 participants, eg, survivors, policy makers, health-care professionals, researchers), including local and international experts, to review and discuss priorities for late effects screening, supportive care interventions, and research in AYAs. The plenary session at the symposium featured the Platinum Study (19-21), a multi-institutional cohort investigation of testicular cancer survivors. The study of testicular cancer survivors represents a unique model for AYA survivorship research given their typical age at diagnosis (18-35 years) (22), treatment with homogeneous platinum-based regimes, and 5-year relative survival rates of 97% (23). The Platinum Study was developed to evaluate and characterize the risk, progression, and health impact of long-term treatment-related toxicities in testicular cancer survivors and, ultimately, propose care guidelines to prevent them. The Platinum Study (19-21), therefore, provides an exemplary model for AYA survivorship research that could be adapted to provide insight into similar issues across other AYA survivor populations.

A recurring concern expressed throughout our symposium was that large-scale AYA-focused research was urgently needed to better understand, screen for, and prevent and/or treat late effects in AYAs. We convened an expert panel from our symposium with the goal of reviewing the specific clinical and research priorities for late effects screening and care in AYAs that were identified by our symposium’s attendees as being among the most frequent, concerning, and actionable for frontline care providers and researchers. Specifically, the aims of this overview are to summarize and discuss the evidence surrounding the symposium’s stakeholder-identified priorities for late effects screening (ie, second cancers, CVD and related risk factors, endocrinopathies, and neurocognitive impairments) and survivorship care (ie, exercise-based prevention and treatment strategies) in AYAs and to promote an agenda for AYA-focused research to address the current knowledge gaps.

## Priorities for Late Effects Screening and Care in AYAs

**Figure 1.**
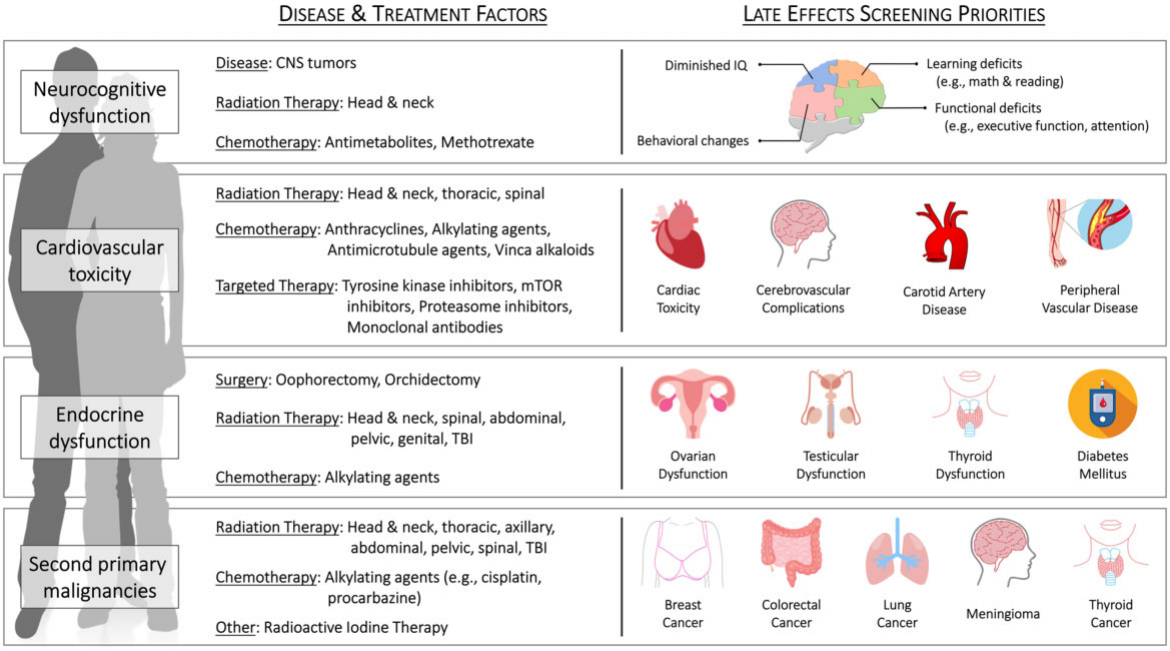
Cancer-related causes of, and screening priorities for, late effects in adolescent and young adult cancer survivors. CNS = central nervous system; mTOR = mammalian target of rapamycin; TBI = total body irradiation.

Our expert panel consolidated the most relevant guidelines from pediatric and adult populations, including the Children’s Oncology Group (COG) (8), National Comprehensive Cancer Network (18), American Society of Clinical Oncology (14,15), and International Late Effects of Childhood Cancer Guideline Harmonization Group (CCGHG) (16,17), into a single set of practical preliminary late effects screening and care guidelines for AYAs (see Figure 1 and Boxes 1-4 for outline and details of late effects screening and management). A focused discussion of the emerging role of exercise therapy in AYA survivorship is included in our overview because it was appraised by the symposium’s stakeholders to be the most promising adjunct therapy to prevent and treat a range of late effects in AYAs and, therefore, a high priority for survivorship care.

Box 1 Recommendations for second malignancy screening and management in AYA cancer survivors^a^

**Colorectal cancer**

**Indication**

- Exposure to abdominal or pelvic RT [≥20 Gγ (18)], spinal RT (lumbar, sacral, whole), or TBI (8)
- Alkylating agents [eg, cisplatin, procarbazine (27,28)]

**Screening**

- **Initiation:**
  Beginning 5 years after RT exposure or at 30 years of age [whichever occurs last (5,8,18)]
  Selected screening approach based on informed decision making between patient and provider (8)
  For patients at high risk because of personal or family history and/or hereditary syndromes predisposing to colorectal cancer, more intensive and earlier screening recommended (8)
- **History:**
  Baseline assessment of personal and/or family history of familial adenomatous polyposis, hereditary nonpolyposis colon cancer, lynch syndrome, inflammatory bowel disease, ulcerative colitis, gastrointestinal malignancy, and adenomatous polyps (8)
- **Testing:** ^b^
**Structural examinations - Preferred:** Colonoscopy [gold standard; every 5 years (8,18)]
**Stool-based tests:** Multitarget stool DNA test (preferred alternative to colonoscopy; every 3 years); **Alternatives:** Fecal immunochemical test (yearly); High-sensitivity, guaiac-based fecal occult blood test [yearly (8,18)]

**Intervention**

- **Medical:**
  Gastroenterology, surgery, and oncology consultation as clinically indicated (8)
- **Behavioral:**
  Refer to exercise specialist and dietician for education and optimizing exercise and dietary behavior to manage long-term risk (101,118)

**Breast cancer**

**Indication**

- Exposure to thoracic RT, axillary RT, and TBI (8,18)

**Screening**

- **Initiation:**
  Beginning 8 years after RT exposure or at age 25 years whichever occurs last (8,18)]
  Women treated with chest RT between 10 and 20 Gγ may participate in shared decision making with their physician about preferred screening approaches (18)
- **History: **
  Baseline assessment of personal and/or family history of *BRCA1*, *BRCA2*, *ATM* (ataxia-telangiectasia mutated) and *p53* mutations (8)
- **Testing:**
  Yearly magnetic resonance imaging with or without mammography (5,8,18)

**Intervention**

- **Medical: **
  Surgery and oncology consultation as clinically indicated (8)
- **Behavioral:**
  Refer to exercise specialist and dietician for education and optimizing exercise and dietary behavior to manage long-term risk (101,118)

**Thyroid Cancer**

**Indication**

- Exposure to RT that includes the thyroid gland [eg, head and/or brain, neck, spine (cervical, whole) or TBI (8,16,18)]

**Screening**

- **Initiation:**
  ≥5 years after RT (8,16)
- **Physical:**
  Thyroid assessment, including neck palpation (1 to 2 years), TSH and free thyroxine [T4; 1-2 years (8, 16, 18)], and fine needle aspiration as clinically indicated (8)
- **Imaging: **
  Ultrasonography examination (3-5 years) to evaluate palpable nodules (8) and to detect clinically impalpable tumors^c^

**Intervention**

- **Medical: **
  Endocrine and surgical consultation for further management (8)

^a^AYA = adolescent and young adult; RT = radiation therapy; TBI = total body irradiation; TSH = thyroid-stimulating hormone

^b^All positive tests should be followed up with a timely colonoscopy.

^c^No evidence of benefit to support imaging over palpation (16).

Box 2 Recommendations for cardiovascular toxicity screening and management in AYA cancer survivors^a^

**Indication**

- Exposure to cardiotoxic therapies, including the following (8,14,18):

***High-risk patients***:

- High-dose anthracycline chemotherapy [≥250 mg/m^2^ of doxorubicin (14,18); ≥600 mg/m^2^ epirubicin (14)]
- Thoracic RT 20 Gγ or higher [risk for CAD (18)], 30-35 Gγ or higher [risk for cardiomyopathy (14)], or 35 Gγ or higher [valvular heart disease (18)] with the heart within the treatment field
- Combined therapies including lower-dose anthracycline (<250 mg/m^2^ of doxorubicin, <600 mg/m^2^ epirubicin) with lower-dose chest RT 15-30 Gγ (14,18) (for cardiomyopathy) with the heart within the treatment field
- Lower-dose anthracycline (<250 mg/m^2^ of doxorubicin, <600 mg/m^2^ epirubicin) or trastuzumab alone and presence of any of the following cardiovascular risk factors (14):
  Multiple cardiovascular risk factors (≥2 risk factors), including smoking, hypertension, diabetes, dyslipidemia, and obesity following the completion of therapy (14)
  Compromised cardiac function (eg, borderline low LVEF [50% to 55%], history of myocardial infarction, moderate to severe valvular heart disease) at any time before or during treatment (14)
  Lower-dose anthracycline (<250 mg/m^2^ of doxorubicin, <600 mg/m^2^ epirubicin) followed by trastuzumab (14)

***Low-to-moderate-risk patients***:

- Doxorubicin less than 250 mg/m^2^ and less than 15 Gγ of RT with potential impact to the heart^b^
- Only 15 Gγ or higher and less than 35 Gγ of RT with potential impact to the heart^b^

**Screening**

- **Initiation:**
  Early into survivorship period
  Consider cardiology consultation in patients 5-10 years after exposure to 35 Gγ or higher of chest RT (18)
- **Screening Targets:**
**Anthracycline** **or RT exposure:** 1. Arrhythmia and 2. cardiomyopathy (8)
**RT exposure only:** 1. Atherosclerotic CVD; 2. pericardial disease; and 3. valvular disease (8)
- **History:**
  Baseline history and ongoing assessment of cardiovascular risk factors in survivors who received potentially cardiotoxic treatments (14) and assessment for symptoms of chest pain, dyspnea on exertion, orthopnea, palpitations, and abdominal symptoms [eg, nausea, vomiting (8)]
- **Physical:**
**Anthracycline exposure or RT exposure**—Ongoing assessment of blood pressure, signs of heart failure, auscultation for murmurs
- **Testing:**
  Asymptomatic survivors considered to be at increased risk for developing cardiac dysfunction following the completion of cancer-directed therapy may be offered an ECHO workup between 6 and 12 months posttreatment (14).
  Survivors identified with asymptomatic cardiac dysfunction during routine surveillance should be referred to a cardiologist or health-care provider with cardio-oncology expertise for further assessment and management (14)
  Electrocardiogram (including evaluation of QTc interval in persons exposed to RT dose ≥15 Gγ) and repeat as clinically indicated (18)
  Screening recommendations for vascular disease are undefined
**High-risk patients:** Patients at high risk for cardiomyopathy or valvular heart disease as defined above should be screened via ECHO every 2-5 years (8,18)
**Low-to-moderaterisk patients:** Patients who received treatment with potential impact to the heart should be screened via ECHO every 5 years (8)

**Intervention**

- **Counseling:**
  Discuss the 1. benefits of maintaining a heart-healthy lifestyle, including exercise and diet, for CVD risk factor management (8,14) and 2. risks and benefits of exercise (14)
- **Medical:**
  Regular evaluation and management of modifiable cardiovascular risk factors such as smoking, diabetes, hypertension, dyslipidemia, and obesity in those treated with potentially cardiotoxic therapies (8,14,18)
- **Behavioral:**
  Refer to exercise specialist and dietician for education and optimizing exercise and dietary behavior to manage long-term risk (101,118)
**Special considerations for exercise** (8)**:**
    Regular exercise is generally safe and should be encouraged for patients with normal left ventricular systolic function
    Survivors with asymptomatic cardiomyopathy should consult cardiology to define limits and precautions for exercise
    Cardiology consultation may be reasonable to define limits and precautions for exercise for high-risk survivors (ie, those requiring an ECHO every 2 years) who plan to participate in intensive exercise

^a^AYA = adolescent and young adult; CAD = coronary artery disease; CVD = cardiovascular disease; ECHO = echocardiography; LVEF = left ventricular ejection fraction; MRI = magnetic resonance imaging; RT = radiation therapy; TBI = total body irradiation.

^b^Chest RT, abdominal RT, spinal (thoracic, whole) RT, or TBI. TBI included for cumulative dose calculation purposes only; section not applicable to patients who received TBI alone.

Box 3 Recommendations for endocrine complication screening and management in AYA cancer survivors^a^

**Gonadal** (**Ovarian**) **Dysfunction and Failure**

**Indication**

- Exposure to 1. alkylating agents [eg, procarbazine, cisplatin (8)] or 2. pelvic and spinal RT (sacral, whole), or TBI
- Risk of ovarian failure depends on total exposure, age at exposure, and current age

**Screening**

- **Initiation: **
  1 year posttreatment
- **Screening targets: **
  1. Infertility and 2. transient and permanent premature ovarian insufficiency (8)
- **History: **
  Baseline assessment of menstrual history, sexual function (eg, vaginal dryness, libido), menopausal symptoms, and medication use (8)
- **Testing:**
  Yearly follow-up assessment of screening targets (8)
  Follicular-stimulating hormone and estradiol testing in survivors with suspected premature ovarian insufficiency (8)
  Anti-Müllerian hormone test in survivors desiring fertility

**Interventions**

- **Counseling:**
  Discuss the 1. adverse impact of ovarian hormone deficiencies on growth, bone mineralization, CVD, and sexual dysfunction (8) and 2) risks and benefits of hormonal replacement therapy in survivors with ovarian hormone deficiency (8)
- **Medical:**
  Endocrine and gynecology referral for survivors with abnormal menstrual patterns of menopausal symptoms and initiate hormone replacement therapy if clinically indicated and agreed on by survivor (8)

**Gonadal** (**Testes**) **Dysfunction and Failure**

**Indication**

- Exposure to 1. alkylating agents (eg, cyclophosphamide, cisplatin) and 2. pelvic/testicular RT/TBI (8) consider cyclophosphamide equivalent dose of more than 4 g/m^2^; however, any dose can put men at risk (47,56)

**Screening**

- **Initiation:**
  1 year posttreatment
- **Screening targets:**
  1. Infertility and 2. testosterone deficiency and insufficiency (8)
- **History:**
  Baseline and yearly follow-up assessment of sexual function and/or hypogonadism (eg, erections, nocturnal emissions, libido, mood) (8)
- **Testing:**
  Measurement of early morning testosterone concentration if symptomatic
  Endocrinology referral for patients with testosterone deficiency or insufficiency to weigh risks and benefits of hormonal replacement therapy (8)
  Semen analysis and testosterone levels for men desiring fertility

**Intervention**

- **Counseling:**
  Discuss the 1. adverse impact of testosterone deficiencies on growth, bone mineralization, CVD, and sexual dysfunction (8) and 2. risks and benefits of hormonal replacement therapy in survivors with hypogonadism (8)
- **Medical:**
  Endocrine and/or urology referral for survivors with symptoms of testosterone deficiencies and initiate hormone replacement therapy if clinically indicated and agreed on by the survivor (8)

**Thyroid Dysfunction**

**Indication**

- Exposure to 1. head and/or brain RT, neck RT, spinal RT (cervical, whole), or TBI (8); 2. total radiation dose to hypothalamic-pituitary-adrenal axis of 30 Gγ or more (18); 3. radioiodine therapy (I-131 thyroid ablation (8); and 4. thyroidectomy (8)

**Screening**

- **Initiation:**
  1 year posttreatment
- **Screening target:**
  1. Primary hypothyroidism, 2. central hypothyroidism, and 3. hyperthyroidism (8)
- **History: **
  Baseline and lifelong monitoring of signs and symptoms of hypothyroidism (eg, weight gain, cold intolerance, fatigue, dry skin) or hyperthyroidism (eg, weight loss, tremor, anxiety, heat intolerance, palpitations) in at-risk survivors (8)
- **Physical:**
  Yearly assessment for fatigue, height, weight, dry skin, brittle hair, depressed mood, cold intolerance, constipation, and thyroid function via TSH and free T4 (8)
  More frequent screening recommended during periods of rapid growth (8)

**Intervention**

- **Medical: **
  Refer to endocrinologist for ongoing management given risks associated with hormone deficiencies

**Diabetes Mellitus**

**Indication**

- Exposure to abdominal RT or TBI (8)

**Screening**

- **Initiation: **
  1 year posttreatment (delayed onset but priority for early screening and education)
- **Screening target: **
  1. Impaired glucose metabolism and 2. diabetes mellitus (8)
- **History: **
  Symptoms of hyperglycemia (eg, increased thirst, increased urination, weight loss, blurry vision)
- **Physical: **
  Routine (every 2 years) assessment of fasting blood glucose or HbA1c (8) and consider oral glucose tolerance testing for patients with higher radiation exposure (69)

**Intervention**

Discuss obesity-related health risks (8)
**Medical: **
  Endocrine consultation (8) evaluate and treat other comorbid conditions, including dyslipidemia, hypertension, and overweight or obesity (8)
**Behavioral: **
  Refer to exercise specialist and dietician for education and intervention toward exercise and dietary interventions for preventing and managing diabetes (8)

^**a**^AYA **=** adolescent and young adult; CVD = cardiovascular disease; RT = radiation therapy; T4 = thyroxine; TBI = total body irradiation; TSH = thyroid-stimulating hormone.

Box 4 Recommendations for cancer-related cognitive dysfunction screening and management in AYA cancer survivors^a^

**Indication**

- Diagnosis of primary brain tumor or brain metastases; exposure to treatments targeting the brain including head and/or brain RT or TBI, neurosurgery, CNS-directed chemotherapy (91)
- Consider assessment for anyone reporting cognitive difficulties (ie, memory, attention, processing speed, executive functions) affecting return to work or school after systemic cancer treatment [ie, chemotherapy, hormonal therapy, immunotherapy (8,88)]

**Screening**

- **Initiation: **
  Clinical surveillance beginning early into survivorship period
- **History: **
  Educational and/or vocational progress (8,88)
- **Corollary screening targets for adverse psychosocial and quality of life effects (yearly):**
  1. Social withdrawal, 2. relationship problems, and 3. dependent living (8)
- **Corollary screening targets for mental health disorders (yearly): **
  1. Depression, 2. anxiety, 3. posttraumatic stress, and 4. suicidal ideation (8)

**Interventions** [**neurocognitive, psychosocial, and mental health** (8)]

- **Neurocognitive: **
  Comprehensive neuropsychological assessment using a consistent battery of sensitive, standardized tests and questionnaires (88) as clinically indicated for patients with evidence of impaired educational or vocational progress; identify local sources of support and provide information about cancer-related cognitive dysfunction in the absence of accessible clinical neuropsychology services.
- **Counseling: **
  Education and vocational counseling to facilitate school or work transitions for all patients; psychological consultation in patients with emotional difficulties; referral to professional in community or cancer center (psychologist, social worker, occupational therapist, academic counselor) to support acquisition of academic or vocational accommodations or for cognitive or vocational rehabilitation as appropriate.

**^a^** AYA = adolescent and young adult; CNS = central nervous system; RT = radiation therapy; T4 = thyroxine; TBI = total body irradiation; TSH = thyroid-stimulating hormone

### Second Malignancies

Survivors of cancer in their AYA years are at an increased risk of developing secondary cancers caused by their initial cancer treatments (4,5), including, but not limited to, Hodgkin lymphoma (HL), breast cancer (BC), lung cancer, colorectal cancer (CRC), thyroid cancer, and leukemia. In the absence of data documenting the cost-effectiveness or survival benefit for screening for all types of subsequent primary neoplasms (SPN), herein, we focus on a few key SPNs that are related to common exposures (ie, chest radiation) and have some data to justify screening. It is further important to acknowledge that studies reporting SPN may reflect historical and outdated exposures and therefore an inflated absolute risk in AYA survivors. For example, previously used extended field and larger doses of radiation therapy (RT) for HL resulted in a statistically significant increased risk of CVD and SPN compared with the more contemporary use of lower dose, involved node RT (24). As a result, when inferring an individual patient’s risk, it is important to acknowledge RT field and dose (24,25). Overall, SPN risk management considerations in AYA survivors include understanding risk based on past and contemporary exposures, other concurrent risk factors, and appropriate surveillance measures.

CRC is an example of an SPN amenable to screening in the AYA population. Following abdominal and pelvic RT, absolute excess risks of CRC ranges from 24 to 400 per 100 000 person-years (26-28). Alkylating agent exposure, especially procarbazine and cisplatin, is also associated with increased CRC risk (27-29). Notably, colorectal polyps occur at an increased frequency among survivors exposed to abdominal RT, suggesting that these cancers are screen detectable (28,29). Current COG guidelines recommend CRC screening following RT to the abdomen, pelvis, spine, or total body irradiation (TBI) beginning 5 years after exposure or at age 30 years, whichever occurs last (8). A subsequent study found that colonoscopy-based screening for survivors of pediatric cancer exposed to abdominal and/or pelvic RT is most cost-effective if started from 35 years of age, repeated every 10 years, and stopped according to the survivor’s overall health. Compared with no screening, this approach was estimated to prevent 82% of CRC deaths (30). However, although earlier initiation of screening may detect more cases, it is not cost-effective because of low absolute rates at younger ages. See Box 1 for an overview of modality-specific recommendations for screening initiation and frequency.

BC is another SPN that merits screening. Several hormonal modifiers increase BC risk, including ovarian or chest RT within 1 year of menarche, longer duration of endogenous estrogen, and more than 10 years of maintained ovarian function (31). Current BC screening guidelines are informed by the CCGHG (Box 1) (17). Screening is recommended for patients treated with more than 20 Gγ chest RT, beginning at age 25 years or at 8 years following RT, whichever is later. Annual mammography, magnetic resonance imaging (MRI), or both should continue past the age of 50 years, although mammography alone is less sensitive in AYAs because of the increased density of breast tissue compared with that in older women (17). Indeed, the combination of mammography and MRI has been found to be a superior screening approach than either alone (32,33); however, patients should be counseled regarding MRI false-positives (34).

Finally, thyroid cancer following neck RT is worthy of mention (Box 1). Treatment with neck RT has been found to increase the risk of papillary thyroid cancer, although survival rates are excellent after clinical diagnosis (16). Ultimately, a comprehensive physical exam is often adequate; however, sonography may also be used to screen for clinically impalpable cancers. The CCGHG recommends a “shared decision making” model between patients and providers regarding optimal approaches to surveillance, and the COG recommends an annual physical exam (8,16).

Overall, with the exception of perhaps BC, the data supporting appropriate screening for SPNs in patients who are diagnosed with cancer after age 18 years are scant. The longer life expectancy of AYA survivors, however, warrants that oncologists at least be aware of relevant pediatric survivorship data and guidelines to facilitate counseling and support enhanced SPN screening and management in AYAs.

### Cardiovascular Toxicity

CVD is an important contributor to increased morbidity and mortality risks in AYA cancer survivors (6,35,36). The spectrum of CVD in cancer survivors includes, but is not limited to, coronary artery disease, congestive heart failure, cerebrovascular disease, and vascular disease. (37-40). Subclinical and overt CVD present both during therapy and late into survivorship, and the risk increases with older attained age (36). CVD risk is greater than 2-fold higher compared with demographic-matched noncancer controls (6), with the cumulative incidence ranging from 3% to 8% (6,35) over 10-year follow-up. Moreover, mortality risk is more than 8- to 10-fold higher in AYAs who develop CVD vs those without (6,35).

The development of CVD in cancer survivors has been described as a “multiple-hit” process involving preexisting risk factors, direct treatment-related risks, and secondary (eg, behavioral) risk factors (41). Cancer and CVD share multiple traditional [eg, smoking, physical inactivity (42)] and novel [eg, inflammation (43)] risk factors, and it is likely that AYAs present with unrecognized subclinical CVD (44) that is exacerbated by both exposure to anticancer therapies and related changes in health behaviors. [Boxed-text pkaa099-BOX2] Indeed, AYAs with at least 1 additional CVD risk factor are at 1.8- to 3.2-fold increased risk of developing CVD (6). See Box 2 for a summary of anticancer therapies and related risk factors that should be considered when approaching CVD risk management in AYAs.

An overriding concern for CVD risk management in AYA cancer survivors is the recently described cardiovascular care gap (45). In the AYA oncology setting, this care gap stems from the absence of screening guidelines, lack of risk stratification tools that account for the unique cancer- and treatment-related mechanisms of cardiovascular injury, and misconception that CVD risk management may not be important in survivors who may ultimately die from their cancer. This care gap may be particularly harmful for AYAs who are often more concerned about numerous life stage–related priorities (eg, education, careers, family planning) than self-advocacy.

Current CVD risk management guidelines in survivors of childhood and adult cancers are primarily cardiac centric, despite the growing evidence of systemic cardiovascular injury. For primary prevention, when possible, the guidelines (14) recommend avoiding or minimizing the use of potentially cardiotoxic therapies, lower doses or more tailored approaches to delivering RT, comprehensive CVD risk assessment (including an echocardiogram before initiation of cancer therapy in high-risk patients), management of modifiable CVD risk factors, and consideration of cardioprotective strategies such as dexrazoxane (18). During cancer treatment, routine surveillance with echocardiography or serum biomarkers (eg, troponins) may be appropriate in high-risk patients; however, the optimal screening frequency is not defined. Immediately posttreatment (6-12 months), surveillance in asymptomatic patients should be considered with referral to a cardiac specialist on detection of an abnormality. See Box 2 for summary of CVD-related late effects risk management guidelines from the American Society of Clinical Oncology (14), COG (8), and the National Comprehensive Cancer Network (18).

The risk management guidelines for vascular disease in patients with cancer are less developed. Pretreatment risk factor assessment is recommended in patients about to receive cancer therapy with potential vasculotoxic effects (eg, RT, antimetabolites) (37), including taking a comprehensive CVD history, managing CVD risk factors, educating patients about the risks and symptoms of vascular toxicity, and ongoing monitoring during treatment to enable early recognition of toxicity. Formal long-term risk management guidelines for survivors treated with potentially vasculotoxic cancer therapies are undefined; however, suggestions include a yearly history with physical examination (including ankle-brachial index testing) and a carotid ultrasound every 2 years (37).

### Endocrine Dysfunction

Most data on endocrine dysfunction following cancer have been derived from studies of pediatric survivors (46); although, there is mounting evidence of an increased risk of endocrinopathies in AYAs. The most common endocrinopathies in AYAs are caused by gonadal and thyroid dysfunction and metabolic changes leading to diabetes (Box 3).

The gonads, both ovaries and testes, are particularly vulnerable to the effects of alkylating agents (47) and infradiaphragmatic and pelvic radiation (15,48). Doses of 2 Gγ or more have been shown to impair gonadal function in men and women (15,48). In women, the spectrum of disease includes premature ovarian insufficiency to acute and reversible ovarian failure, the risk proportional to chemotherapy or RT dose, and increasing age at exposure (49,50). For example, women treated for BC aged younger than 40 years have an incidence of premature ovarian insufficiency between 23% and 77% (51), whereas women receiving the highest tertile of procarbazine for lymphoma have a 65% cumulative risk of early menopause (52). Men have a higher risk of infertility rather than hypogonadism given differing susceptibility to damage from chemotherapy and RT between germ cells (ie, sperm-producing cells) and Leydig cells (ie, testosterone-secreting cells). The COG recommends screening symptomatic survivors who received pelvic RT, TBI, or alkylating agents for hypogonadism (8). Because screening is based on symptoms, clinicians need to be well aware of the manifestations of hypogonadism to consider hormone replacement therapy. This is particularly important for women where there is evidence that untreated premature ovarian insufficiency contributes to reduced quality of life, CVD, neurocognitive decline, and osteoporosis (53-55). Recently, a risk stratification model was published that summarizes currently available data for infertility risk for pediatric and adolescent cancer survivor, useful in both the clinical setting and for promoting research in this area (56). 

Thyroid disease is a common late effect of treatment in AYAs given the radiosensitivity of the thyroid gland. Thyroid disease can manifest as primary (most common) or central hypothyroidism, hyperthyroidism, thyroid nodules, and cancer, and the risk for thyroid dysfunction persists even 20 years posttreatment (57). Jensen et al. (58) conducted the only population-based study to date exploring the risk of endocrine late effects in AYA survivors (n = 32 548) and reported that thyroid disease was the leading reason for a hospital visit, in particular with treatment for HL. Other studies, not specifically in AYA survivors, have reported hypothyroidism in up to 50% of HL survivors as well as a strong dose-response relationship between neck radiation and risk of hypothyroidism (59). The COG guidelines recommend lifelong screening for thyroid hormone dysfunction with laboratory tests following RT treatment to the head and neck, spine, or TBI (8). An annual physical exam of the thyroid is the only recommended screening modality for thyroid nodules and cancer (discussed in “Second Malignancies” section).

Diabetes is an emerging late effect of cancer treatments. Pediatric survivors have a 60%-80% overall increased risk of diabetes (60-62), and survivors of AYA cancers may also be at increased risk. Jensen et al. (58) also reported a 29% increased risk of diabetes in AYAs compared with the general population and found that diabetes was one of the leading reasons for hospital visits. Studies in specific AYA cancers have also reported increased diabetes risk among HL (63) and testicular cancer survivors treated with para-aortic RT (64). The pathogenesis of diabetes in these populations is largely related to pancreatic and adipose tissue toxicity from abdominal RT and chemotherapy, leading to changes in pancreatic function and insulin resistance (65-67). Traditional lifestyle factors including physical inactivity and poor diet may also contribute to increased diabetes risk in survivors (68). Current COG guidelines recommend screening survivors who received abdominal RT or TBI with glycated hemoglobin (HbA1c) and/or fasting glucose measurements every 2 years (8). However, these pediatric-specific recommendations may not be suitable for AYAs given differences in age and treatment exposure, and there is emerging evidence that HbA1c and fasting glucose alone may be inadequate for identifying diabetes following abdominal RT and TBI (69). Ultimately, preventing and treating diabetes is an important initiative for improving long-term outcomes in AYAs given their high burden of CVD (35) as well as evidence that diabetes further increases the risk of major cardiac events, independent of cancer therapy–related cardiac risk factors, in AYAs (6).

### Neurocognitive Effects

Cancer and treatments can adversely impact neurocognitive functions. The neurocognitive sequelae of cancer-related cognitive dysfunction (CRCD) include decrements in attention, memory, processing speed, and executive functions (70). CRCD research has been conducted primarily in pediatric survivors (70-72) and in older women with BC (73,74), although CRCD has recently been documented in other cancers common in AYAs [eg, ovarian (75) and testicular (76-78)]. Both immature (71,79) and aging (80) brains are vulnerable to cancer treatment–related injury. AYAs may be particularly vulnerable to CRCD because the frontal lobes continue to develop throughout young adulthood (81), and frontal lobe injury alters maturation of executive functions (82).

Few studies have examined CRCD in AYAs to date (10). About 30% of AYAs report problems completing higher education or maintaining full-time employment several years after diagnosis, and more than 30% report problems with attention, memory, and processing speed (11,83-85). CRCD may underlie these problems. Indeed, cognitive symptoms in adult survivors of cancers diagnosed in early AYA years are associated with poorer academic, vocational, and social outcomes many years posttreatment; those diagnosed with brain tumors or treated with cranial RT have the poorest outcomes (11). Cancer-related disruptions in psychological adjustment and emotional distress can further impact cognitive performance in AYAs (86). In the short term, AYA survivors of noncentral nervous system cancers do not show the same patterns of cognitive decline reported in older adult survivors during the first year postdiagnosis; however, those treated with chemotherapy are at increased risk for persistent emotional distress (87). Whether neurocognitive effects of cancer treatment emerge later in AYAs, placing them at risk for accelerated aging, remains to be examined. In the interim, addressing neurocognitive and psychosocial outcomes in AYAs is critical to ensure acquisition of key developmental milestones of this life stage.

Guidelines developed for pediatric (8) and adult (88) cancer survivors to address these outcomes are also relevant for AYAs (Box 4). Specifically, monitoring survivors for psychosocial and neurocognitive concerns during and after treatment is necessary to facilitate return to school and work. Routine monitoring and providing psychological interventions for emotional distress are needed to address the unique psychosocial issues associated with this life stage (86,89). Moreover, neurocognitive screening should be conducted for survivors at risk for adverse neurocognitive outcomes (those with primary brain tumors or metastases and treated with cranial RT and central nervous system–directed chemotherapy) using a consistent battery of sensitive, standardized tests, as previously recommended (88,90). Comprehensive neuropsychological assessments may also be warranted for those who continue to struggle with reentry to school or work and can be offered at 2- to 3-year intervals in response to suspected changes in cognition (91) or at key transition times such as prior to postsecondary education or changing careers (92). Limited availability and costs of clinical neuropsychology services and inconsistent reimbursement by private insurers pose challenges to implementation of these recommendations. Nonetheless, at minimum, identifying local sources of support (eg, university counseling services, employee support programs) and providing those sources with information about CRCD may be helpful. An adult educational and vocational counseling program to support the transition of pediatric survivors from high school to college and/or the labor force has already been developed in Ontario (92). Similar programming is recommended to address transition issues in AYAs, including reintegration into school or work.

In summary, even modest compromise of cognitive functioning can have a meaningful impact on psychological well-being affecting education and occupational attainment (84), with lifelong implications. Systematic research is needed to further characterize CRCD in AYAs and inform the development of interventions that alleviate psychosocial and cognitive sequelae, so that survivors achieve their full potential.

## The Role of Exercise Testing and Prescription in AYA Survivorship

Many cancer-related sequelae experienced by AYAs have complex etiologies involving multiple overlapping mechanisms, making them difficult to prevent and treat. This complexity, however, creates a strong rationale to explore multitargeted prevention and treatment strategies, like exercise.

For example, CRF (ie, cardiorespiratory fitness, measured as VO_2peak_ or Metabolic equivalent of tasks) is assessed via symptom-limited maximal cardiopulmonary exercise test and reflects the integrative capacity of the cardiovascular system to transport oxygen from the environment to skeletal muscle mitochondria to produce energy (93). CRFis one of the most robust predictors of cardiovascular health and longevity across healthy and clinical populations (93), and impaired CRFis emerging as an important marker of cancer-related cardiovascular injury and mortality risk in oncology (94,95). CRFis reduced in certain AYA (7) and adult (96) cancer survivor groups because of direct treatment-related (eg, cardiomyocyte injury) and secondary lifestyle-related (eg, physical inactivity, obesity) factors and it may not recover in the years following treatment [eg, in BC survivors (96)]. In oncology, low CRFis associated with increased rates of treatment-related toxicities, greater symptom burden, and increased all-cause, CVD-, and cancer-specific mortality risk (95,97). However, evidence from noncancer clinical populations shows aerobic exercise training helps prevent acute cardiovascular injury (98), improves organ-specific (99) and coordinated cardiovascular function [eg, CRF(100)], and reduces mortality risk (93); thus, it may similarly benefit cancer survivors.

Indeed, exercise may be effective in reducing cancer-specific and all-cause mortality (101), cancer recurrence (101), and preventing and treating cancer-related sequelae AYAs commonly experience, including CRFimpairment (97), metabolic and endocrine dysfunction (102), cardiovascular toxicity (94,103), psychological distress (101), and cognitive impairments (104). Exercise may also improve other important outcomes in AYAs, as demonstrated in other survivor groups [eg, sarcopenia and skeletal muscle dysfunction in younger and older BC survivors (105)]; yet, this presumption has not been confirmed in AYAs. In fact, remarkably little evidence supporting the benefits of exercise in oncology originates from AYA-focused research. Epidemiological data in AYAs consistently suggest that participation in vigorous intensity physical activity, in particular, is associated with decreased morbidity (20,106) and mortality (107) risk. The findings from randomized controlled trials (RCTs) of exercise in AYAs, however, are mixed. To date, most RCTs in AYAs have tested self-directed, home-based interventions targeting the achievement of the general cancer exercise guidelines (108,109) and have failed to produce meaningful improvements in measured behavioral, physical, and psychosocial outcomes, relative to controls (110-112). Conversely, a recent RCT of individually tailored high-intensity aerobic interval training in 63 testicular cancer survivors reported statistically significant improvements in CRF(113), mental health–related quality of life, fatigue, and self-esteem (114); and reductions in the prevalence of modifiable CVD risk factors and CVD risk (113). Rigorous research evaluating the safety and impact of theoretically sound exercise interventions (ie, adherent to the principles of exercise prescription) in AYAs is urgently needed.

Finally, exercise was recently adopted as a standard of cancer care in Australia (115) and will likely similarly be adopted in North America. Exercise engagement may be particularly important for AYAs given their higher 5-year survival rates (82.5%) and the greater potential for years of productive life lost per individual than people diagnosed after the age of 40 years (116). Exercise may be among the most effective single intervention approaches to address health concerns in AYAs, despite the current lack of grade A evidence supporting it. Exercise prescriptions should be specific (ie, targeted to an outcome), individualized (ie, tailored to a person’s fitness level), and progressed (ie, systematically increasing physiologic demand) to safely optimize adaptations (117); and, the lack of benefits noted previously in AYA trials (110-112) likely reflects inadequate consideration of these principles (117). Until more rigorous evidence is available, practitioners are encouraged to adopt current clinician guidelines (118) for exercise screening, advisement, and referral to appropriate community- and hospital-based resources to facilitate exercise engagement in AYAs.

## Current Limitations and Recommendations for AYA Survivorship Research

AYA survivorship is increasingly being recognized by leading cancer care entities in Canada (119) and the United States (18) as a priority for specialized research. However, the recommendations within the current follow-up guidelines (8,14,16-18) have not been validated and rely heavily on consensus statements in which there is an unproven assumption that because a given late toxicity occurs, screening for it must be worthwhile. Readers should, therefore, interpret the recommendations within this overview [and the guidelines that informed them (8, 14,16-18)] with caution. There is a critical need to conduct research that challenges these assumptions and improves the rigor of the evidence underlying late effects screening and follow-up guidelines in AYAs according to best practice criteria for disease screening (120,121). Moreover, research that tests tailored and scalable strategies to prevent and manage late effects in AYAs is remarkably scant. The Platinum Study is an exemplary model for rigorous, prospective, multi-institutional survivorship research (19-21), and well-funded initiatives are urgently needed to advance the care for other common, and similarly vulnerable, groups of AYAs (eg, lymphoma and BC). See Table 1 for a summary of recommended AYA survivorship research priorities.

**Table 1.** Research priorities for late effects screening and management in AYA cancer survivors^a^

Research domain	Research priorities
Risk	Understanding the biological and behavioral determinants of cancer- and treatment-related late effects (hereafter, late effects) to clarify what differentiates those who develop late toxicity from those who do not
Screening and risk stratification	Identifying and validating novel biomarkers (eg, CRF) to augment late effects screening and risk stratification in AYA cancer survivors Validating current and emerging strategies to screen for the spectrum of late effects in AYA cancer survivors according to best principles and practices criteria for disease screening (120,121) Defining discrete subgroups of AYAs at moderate-to-high risk of specific late effects and characterizing the nature and mechanisms of injury and/or dysfunction within these subgroups Improving the uptake of appropriate screening where the evidence is good (eg, early initiation of breast cancer screening after chest RT)
Intervention	Developing novel, and refining current, intervention strategies to optimize AYA engagement Conducting rigorously designed trials testing the effects of theoretically grounded interventions targeting the unique risk factors and mechanisms underlying the subgroup-specific sequelae
Follow-up	Establishing best practices and models of long-term follow-up care for AYAs
Evidence quality	Evaluating the rigor and quality of the current screening guidelines (8, 14–18) and the studies that have informed them

a AYA = adolescent and young adult; RT = radiation therapy; CRF= cardiorespiratory fitness.

## Conclusion

The current scarcity of AYA-specific data on late effects screening and management limits the opportunities for more comprehensive evidence review; thus, we highlighted that which may be the most actionable for frontline health-care providers and impactful for patients. Exercise is a multitargeted behavioral intervention strategy that represents an accessible, efficacious, and patient-preferred therapeutic approach to reduce the risks of late effects in AYAs. Research evaluating the validity of current assumptions and generating new knowledge to develop AYA-specific screening and care guidelines is urgently needed. To this end, the success of collaborative prospective cohort investigations, like the Platinum Study, suggests that similar AYA-focused initiatives may well be feasible and high yielding.

## Funding

This review was not funded.

## Notes

**Role of the funder: **Not applicable.

**Disclosures:** The authors report no conflict of interest.

**Author contributions**: Conceptualization: AG, LM; Writing—Original Draft: SA, JH, IL, KE, PT, AG; Writing—Review & Editing: All authors.

**Acknowledgements:** We thank the attendees of The AYA Survivorship Symposium: A New Vision (March 2019) for their contributions to identifying the priorities discussed within this review. We also thank the Michael Kamin Hart Family for sponsoring the symposium and whose dedication to AYA oncology is without limits

## Data Availability

All relevant data is provided within this manuscript.
